# Placental morphological features of small for gestational age preterm neonates born to mothers with pregnancy-induced hypertension

**DOI:** 10.3389/fped.2023.1093622

**Published:** 2023-03-21

**Authors:** Yu Zhang, Hui-Hui Zeng

**Affiliations:** Department of Neonatal Intensive Care Unit, Beijing Obstetrics and Gynecology Hospital, Capital Medical University, Beijing Maternal and Child Health Care Hospital, Beijing, China

**Keywords:** pregnancy-induced hypertension, preterm, small for gestational age, placenta, morphological feature, precise prenatal pre-evaluation

## Abstract

**Introduction:**

Small for gestational age (SGA) neonates are often born to mothers with pregnancy-induced hypertension (PIH). Here, we aimed to explore the morphometric characteristics of the placenta during the perinatal period associated with SGA risk in mothers with PIH and identify the risk factors related to SGA.

**Methods:**

The medical records of 134 neonates born between 28- and 32-weeks’ gestation to PIH mothers were retrospectively analyzed. Placental morphology and umbilical cord (UC) length were compared between the SGA and appropriate for gestational age (AGA) groups.

**Results:**

The placenta of the SGA group had a shorter major (15.00 vs. 18.00 cm; *z* = −6.04, *p *< 0.01) and minor placenta axes (13.00 vs. 15.00 cm; *z* = −4.59, *p *< 0.01), lower weight (300.00 vs. 420.00 g; *z* = −7.21, *p *< 0.01), smaller volume (282.00 vs. 396.00 cm^3^; *z* = −5.00, *p *< 0.01), and smaller area (141.00 vs. 212.00 cm^2^; *z* = −5.96, *p *< 0.01) than the AGA group. The UC was significantly shorter (39.00 vs. 44.00 cm; *z* = −3.68, *p* < 0.01). Short placental major axis [*p *= 0.03; odds ratio (OR): 2.16; 95% confidence interval (CI): 1.84 − 2.63] and low placental weight (*p *< 0.01; OR: 2.68; 95% CI: 2.66 − 2.70) were independent risk factors for SGA in premature newborns of PIH mothers.

**Discussion:**

A major axis shorter than 15.5 cm or placental weight lower than 347.50 g at birth was related to a greater risk of SGA infants born to PIH mothers. As a predictor in prenatal ultrasound, the major axis is more helpful for precise prenatal pre-evaluation of vulnerable SGA preterm neonates with PIH mothers.

## 1. Introduction

Immature development of multiple organs has been reported in preterm neonates, with mortality rates higher than that in term neonates (1, 2). Small for gestational age (SGA) is commonly defined as a weight below the 10th percentile of the average value for the gestational age or less than two standard deviations. The mortality of SGA preterm neonates is 15 times higher than that of neonates who are SGA or preterm alone (3). In SGA preterm infants, neonatal complications, such as bronchopulmonary dysplasia, sepsis, hypoglycemia, necrotizing enterocolitis, polycythemia, and prolonged hospitalization, are more common than in appropriate for gestational age (AGA) infants (4, 5). These findings were associated with increased morbidity and mortality among premature SGA newborns, suggesting the importance of precise prenatal pre-evaluation and that more attention should be paid to this vulnerable population before and after delivery (6, 7).

Pregnancy-induced hypertension (PIH) is a common and serious perinatal complication often leading to the birth of premature SGA neonates (8, 9). PIH comprises gestational hypertension, preeclampsia, preeclampsia superimposed on chronic hypertension (CH), and CH (8). Panaitescu et al. conducted a cohort study of 74,226 pregnancies, and observed that the SGA delivery rates in pregnancies with CH and preeclampsia alone were separately twice and ten times as high as in those without CH (10).

The placenta supports intrauterine life and fetal development through its nutritional, immunologic, and endocrine functions (11). Existing studies on morphological features of the placenta associated with SGA mainly focused on premature neonates and only some on term neonates (11, 12). However, few studies have focused on SGA-related morphological features of placentas that are specific to PIH or in the perinatal period. Therefore, the primary objectives of the present research were to explore the exact morphometric characteristics of the placenta during the perinatal period associated with SGA risk in mothers with PIH and identify the risk factors related to SGA. Refined prenatal pre-evaluation, early recognition of SGA status, timely intervention to prevent associated complications, and individualized management, including fluid, electrolyte, and respiration management, should be considered before and after the delivery of vulnerable premature SGA newborns of mothers with PIH.

## 2. Materials and methods

### 2.1. Study design and population

This preliminary retrospective case-control research was performed in the neonatal department of a level 3 hospital in Beijing from January 2018 to December 2020. Ethical clearance (2021-KY-106-01) was obtained from the institutional ethical committee. Preterm SGA neonates with a gestational age (GA) of 28–32 weeks, born to mothers with PIH, were enrolled in the SGA group. Preterm AGA infants without intrauterine growth retardation, also born to mothers with PIH, were consecutively enrolled in the control group till two groups showed an even distribution for GA at birth. The exclusion criteria included (a) multiple pregnancies; (b) family history of inherited metabolic diseases; (c) abnormal test results in non-invasive detection of fetal chromosomal aneuploidy in maternal blood; (d) chromosomal or metabolic gene abnormalities diagnosed by chorionic villus sampling; (e) abnormality in deafness gene and hearing screening; (f) congenital malformation in the newborn; and (g) incomplete clinical records.

SGA is defined when a newborn's birth weight is less than two standard deviations or below the 10th percentile of the mean for GA based on defined standards (local birth weight standards) for males and females (12–14). Preterm birth is commonly defined as live birth occurring before the 37th week of pregnancy (14). PIH was defined as the presence of pre-eclampsia or hypertension in pregnancy, according to the guidelines of the Chinese Society of Obstetrics and Gynecology (15).

### 2.2. Data collection

Neonatal gender, length, weight, GA at birth; placental weight, length (major axis), width (minor axis), thickness, and length of the umbilical cord (UC); and mother's age at birth, mode of delivery, number of pregnancies and deliveries, perinatal complications (PIH, pregnancy-induced diabetes, thyroid dysfunction, and fetal distress) were obtained from the patients’ medical records. Methods of these measurements were referenced to the description by Barker et al. (16). Briefly, the maximal diameter (major axis) of the surface and the lesser diameter (minor axis) bisecting it at right angles were measured with a scale. Two diameters were measured and used to describe the placental surface because it was more oval than circular. The maximum thickness of the placenta taken from the basal plate and perpendicular to the long axis of the placenta was measured as the placental thickness. The length of the umbilical cord was measured with a scale when it was in a straight line without tensile deformation. Placental area and perimeter were calculated using an online calculator (17). All measurements were conducted by the same experienced obstetrics team and completed within 10 min after neonates were born. In all cases, resuscitation and stabilization had priority over all other procedures.

### 2.3. Sample size calculation

No *a priori* sample size was calculated and all the eligible participants during the study period were included.

### 2.4. Statistical analysis

Median (interquartile range) was used to show quantitative data. Absolute frequency (percentage) was used to show dichotomous data. Mann − Whitney *U* test was used to compare non-normally distributed continuous variables (maternal age, gravidity, parity, neonatal GA, birth weight, birth length, placental weight, major axis, minor axis, thickness, perimeter, area, and volume) between SGA and AGA groups. Dichotomous data was compared between the two groups using the Chi-square or Fischer's exact test. Correlations between neonatal birth weight and the placental major axis, minor axis, area, volume, weight, and UC length were detected using Spearman rank correlation analysis. Potential risks and independent risk factors for growth retardation in preterm neonates with mothers of PIH were detected by univariate and multivariate logistic regression analyses, separately. The predictive accuracy of placental weight or major axis to estimate SGA preterm neonate birth to a mother with PIH was determined using receiver operating characteristic (ROC) curve analysis. Data was analyzed using SPSS Statistics for Windows, Version 21.0 (IBM, Armonk, NY). A two-sided *p*-value of <0.05 was considered statistically significant.

## 3. Results

### 3.1. Study population

Between January 2018 and December 2020, 2,143 premature infants were hospitalized in the neonatal department of our hospital. Eighty-nine preterm neonates born between 28- and 32-weeks’ gestation to mothers with PIH were diagnosed with SGA. Sixty-five premature SGA infants were included in the SGA group according to the exclusion criteria. Sixty-nine premature AGA infants with matched GA, born to mothers with PIH were enrolled into the AGA (control) group, until GA of neonates in each group was evenly distributed. Hospitalization records of 134 premature Chinese infants and their mothers were complete for retrieval.

Table 1 shows patients’ clinical and demographic features. Gender, GA, or fetal distress occurrence in neonates of the SGA and control groups showed no statistical difference (*p *> 0.05). The neonatal birth length and weight in the SGA group were significantly lesser than in the AGA group (both *p*-values < 0.01). The rate of fetal distress was higher in the SGA preterm neonates than in the control neonates [30/65 (46.15%) vs. 19/69 (27.54%); *p *= 0.03]. The natural childbirth rate in the SGA group was significantly lower than in the AGA group [3/65 (4.62%) vs. 12/69 (17.39%), *p *= 0.01]. Other data regarding maternal information, such as age, gravidity, parity, primiparity, composition of subtype hypertension, gestational complications (pregnancy-induced diabetes and hypothyroidism, among others), and occurrence of abnormal amniotic fluid, showed no marked difference between both groups.

**Table 1:** Demographic and clinical characteristics of preterm neonates and their mothers.

Variables			SGA group (*n* = 65)	Control (*n* = 69)	*p*-value
Mother baseline	Age		32.00 (27.00–37.00)	33.00 (26.00–40.00)	*p *= 0.20
	Gravidity		2.00 (0.00–4.00)	2.00 (0.00–4.00)	*p *= 0.37
	Parity		1.00 (0.00–2.00)	1.00 (0.00–2.00)	*p *= 0.43
	Primiparity	Yes	45 (69.23%)	45 (65.22%)	*p *= 0.71
	Delivery mode	Vaginal delivery	3 (4.62%)*	12 (17.39%)	*p *= 0.01
	Subtype hypertension	Pre-eclampsia-eclampsia	49 (75.38%)	41 (59.42%)	*p *= 0.46
		Gestational hypertension	4 (6.15%)	7 (10.15%)	*p *= 0.40
		Chronic hypertension	1 (1.5%)	6 (8.70%)	*p *= 0.12
		Chronic hypertension with superimposed preeclampsia	11 (16.92%)	15 (21.74%)	*p *= 0.48
	Gestational complication	Diabetes	14 (21.54%)	19 (27.54%)	*p *= 0.26
		Hypothyroidism	10 (15.81%)	14 (20.29%)	*p *= 0.80
		Other	41 (63.08%)	46 (66.67%)	*p *= 0.46
	Fetal distress	Yes	30 (46.15%)*	19 (27.54%)	*p *= 0.03
	Abnormal amniotic fluid	Yes	15 (23.08%)	11 (15.94%)	*p *= 0.30
Neonatal baseline	Gender	Male	44 (67.69%)	49 (71.01%)	*p *= 0.67
	Gestational age (weeks)		31.00 (29.00–33.00)	31.00 (29.00–33.00)	*p *= 0.95
	Birth weight (g)		1110.00 (825.00–1395.00)*	1530.00 (1012.50–2047.50)	*p *= 0.00
	Birth length (cm)		38.00 (34.00–42.00)*	41.00 (36.0–46.00)	*p *= 0.00

* Statistically significant difference (*p *< 0.05) between gestational age-matched preterm SGA group and control group.

Differences in morphological features of the placenta and UC length between the SGA and AGA groups after birth when compared with the placentas of GA-matched AGA preterm infants of mothers with PIH, the placentas of SGA preterm infants of mothers with PIH were characterized by significantly lower weight (300.00 vs. 420.00 g; *z* = −7.21, *p *< 0.01), smaller volume (282.00 vs. 396.00 cm^3^; *z* = −5.00, *p *< 0.01), smaller area (141.00 vs. 212.00 cm^2^; *z* = −5.96, *p *< 0.01), shorter major axis (15.00 vs. 18.00 cm; *z* = −6.04, *p *< 0.01), shorter minor axis (13.00 vs. 15.00 cm; *z* = −4.59, *p *< 0.01), and shorter UC length (39.00 vs. 44.00 cm; *z* = −3.68, *p *< 0.01) (Table 2). Placental thickness (2.00 vs. 2.00; *z* = −0.25; *p *= 0.80) and perimeter (33.00 vs. 29.30; *z* = −0.53; *p *= 0.60) showed no statistical difference between the SGA and control groups (Table 2).

**Table 2:** Comparison of morphological features of the placenta and umbilical cord length between small for gestational age and appropriate for gestational age groups within 10 min after birth.

Items	SGA group (*n* = 65)	Control (*n* = 69)	*p-*value
Placental major axis (cm)	15.00 (11.00–19.00)*	18.00 (14.00–22.00)	*p *= 0.00
Placental minor axis (cm)	13.00 (9.00–17.00)*	15.00 (13.0–17.00)	*p *= 0.00
Placental thickness (cm)	2.00 (1.90–2.10)	2.00 (2.00–2.00)	*p = *0.80
Placental perimeter (cm)	33.00 (16.55–49.45)	29.30 (8.60–50.00)	*p *= 0.60
Placental area (cm^2^)	141.00 (65.00–217.00)*	212.00 (148.00–276.00)	*p *= 0.00
Placental volume (cm^3^)	282.00 (130. 00–434.00)*	396.00 (210.50–582.00)	*p *= 0.00
Placental weight (g)	300.00 (217.50–382.50)*	420.00 (277.50–562.50)	*p *= 0.00
Umbilical cord length (cm)	39.00 (26.00–52.00)*	44.00 (26.00–62.00)	*p *= 0.00

* Statistically significant difference (*p* < 0.05) between gestational age-matched preterm SGA group and control group.

### 3.2. Predisposing risk factors of growth retardation in preterm neonates of mothers with pregnancy-induced hypertension

Spearman’s test (Table 3) showed that the birth weight of preterm neonates was significantly associated with the placental major (*ρ* = 0.52, *p *< 0.01) and minor axes (*ρ* = 0.46, *p *< 0.01), area (*ρ* = 0.54, *p *< 0.01), volume (*ρ* = 0.51, *p *< 0.01), weight (*ρ* = 0.62, *p *< 0.01), and UC length (*ρ* = 0.37, *p *< 0.01).

**Table 3:** Spearman correlation analysis of morphological indexes related to placenta or umbilical cord with birth weight of neonates.

Items (*n* = 134)		Spearman correlation coefficient (*ρ*-value)	*p-*value
Placental major axis	Birth weight	0.52	<0.01
Placental minor axis	Birth weight	0.46	<0.01
Placental area	Birth weight	0.54	<0.01
Placental volume	Birth weight	0.51	<0.01
Placental weight	Birth weight	0.62	<0.01
Umbilical cord length	Birth weight	0.37	<0.01

Univariate logistic regression analysis (Table 4) showed that the placental major axis (*p *< 0.01; odds ratio (OR) = 1.84; 95% confidence interval (CI): 1.65 − 2.09), minor axis (*p *< 0.01; OR: 1.99; 95% CI: 1.77 − 2.24), weight (*p *< 0.01; OR: 2.67; 95% CI: 2.65 − 2.69), and UC length (*p *< 0.01; OR = 2.58; 95% CI: 2.50 − 2.67) were risk factors for growth retardation in premature infants of mothers with PIH. Multivariate regression analysis showed that short major axis (*p *= 0.03; OR: 2.16; 95% CI: 1.84 − 2.63) and low placental weight (*p *< 0.01; OR: 2.68; 95% CI: 2.66 − 2.70) were independent risk factors for SGA in premature newborns of PIH mothers (Table 3), which associated higher SGA delivery risks with 2.16 times and 2.68 times, respectively.

**Table 4:** Placental and umbilical cord risk factors for small for gestational age in preterm neonates of PIH mothers.

Parameters	Univariate logistic regression		Multivariate logistic regression	
	OR (95% CI)	*p*-value	OR (95% CI)	*p*-value
Placental major axis	1.84 (1.65–2.09)	0.00*	2.16 (1.84–2.63)	0.03*
Placental minor axis	1.99 (1.77–2.24)	0.00*	2.44 (2.02–3.10)	0.34
Placental weight	2.67 (2.65–2.69)	0.00*	2.68 (2.66–2.70)	0.00*
Umbilical cord length	2.58 (2.50–2.67)	0.00*	2.78 (2.67–2.92)	0.28

CI, confidence interval; OR, odds ratio.

* Statistically significant difference (*p* < 0.05).

**Figure 1:**
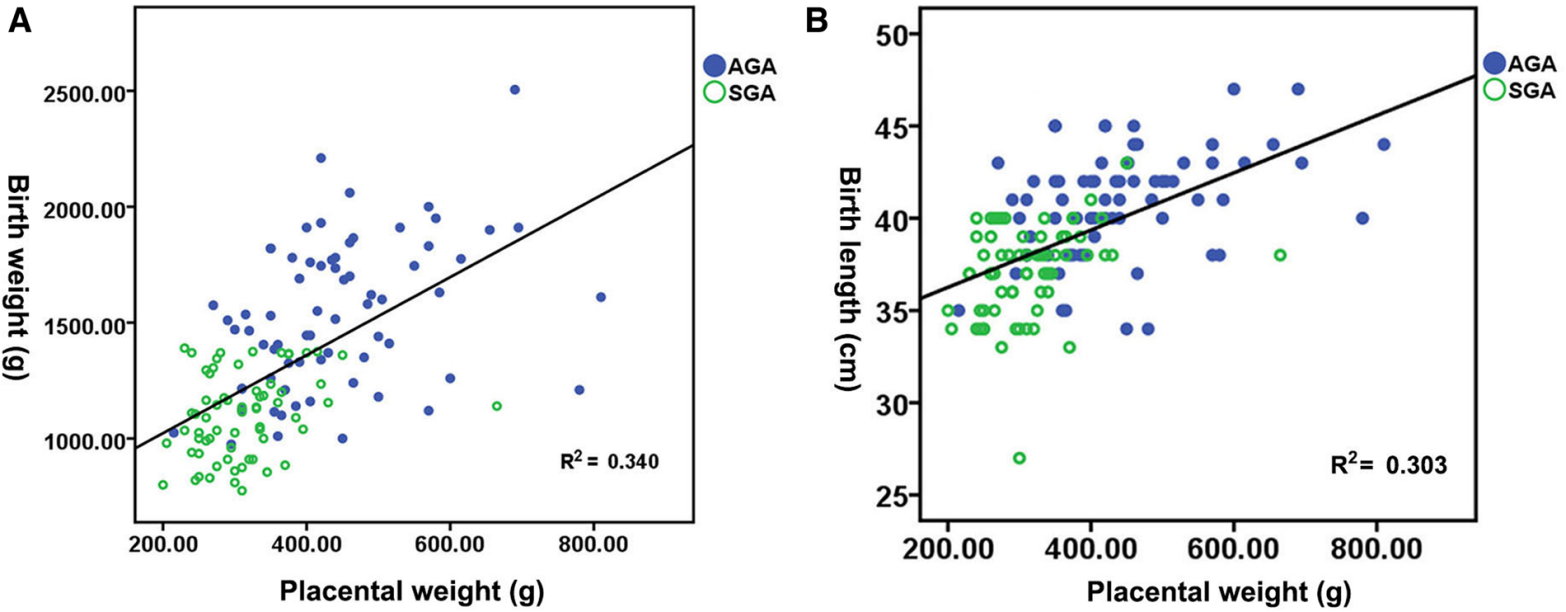
Distribution of birth weight **(A)** and length **(B)** in accordance with placental weight.

**Figure 2:**
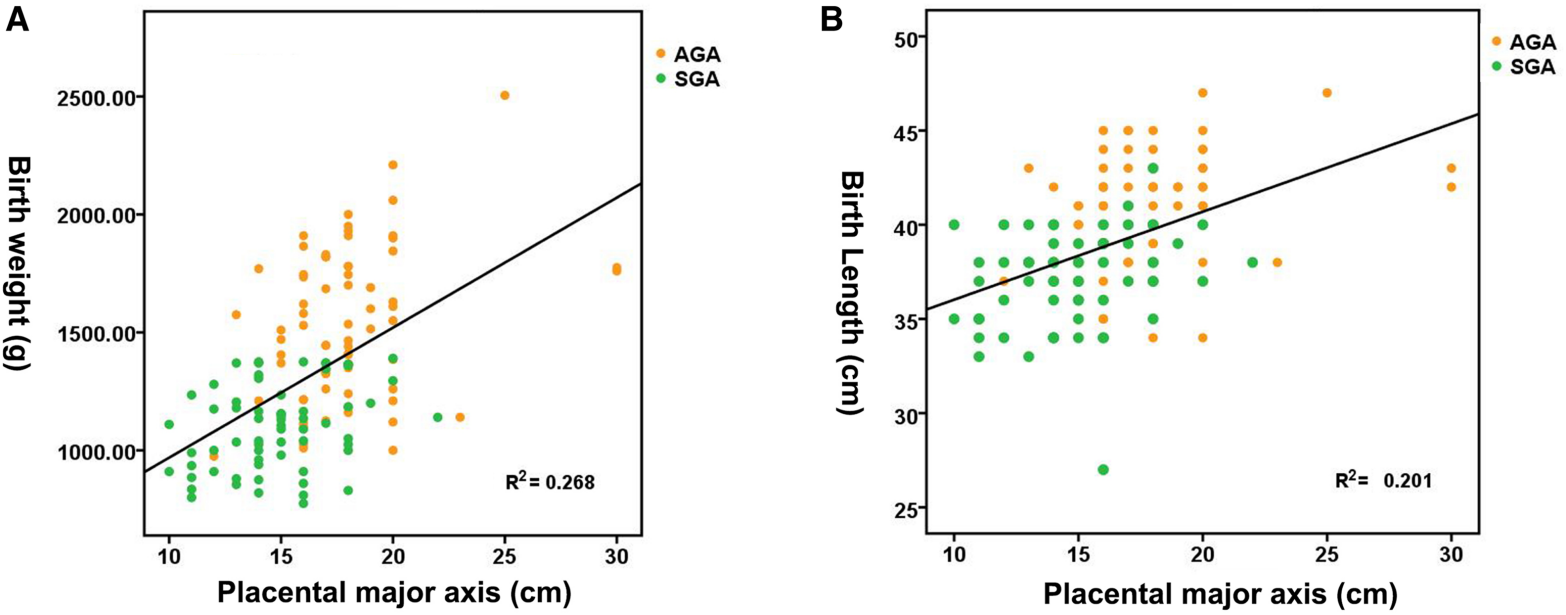
Distribution of birth weight **(A)** and length **(B)** according to placental major axis.

Figure 1A shows the distribution of neonatal birth weight according to placental weight at birth, suggesting a nearly linear state relationship (*R*^2^ = 0.340, *p *< 0.01). The median birth weight increased from 1,020 g for a placental weight of 200 to 2,047.80 g for a placental weight of 810 g. Figure 1B shows the distribution of neonatal birth length by placental weight at birth, demonstrating an almost linear relationship (*R*^2^ = 0.303, *p *< 0.01). The median birth length increases with advancing placental weight from 37.15 cm at a placental weight of 200 g to 49.35 cm at a placental weight of 810 g. Figure 2A shows a significant direct relationship between neonatal birth weight and placental major axis at birth (*R*^2^ = 0.278, *p *< 0.01). The median birth weight increased from 969.10 g for a placental major axis of 10 cm to 2071.30 g for a placental major axis of 30 cm. PIH mothers with shorter placental major axis at birth have a higher risk of giving birth to newborns with lower birth weight. Figure 2B shows a significant direct relationship between neonatal birth length and placental major axis at birth (*R*^2^ = 0.201, *p *< 0.01). The median birth length increases with the advancing placental major axis from 36.05 cm at the placental major axis of 10 to 45.45 cm at the placental major axis of 30 cm. PIH mothers with shorter placental major axis at birth have a higher risk of giving birth to newborns with shorter birth length.

### 3.3. Diagnostic capability of placental weight and major axis in small for gestational age

**Figure 3:**
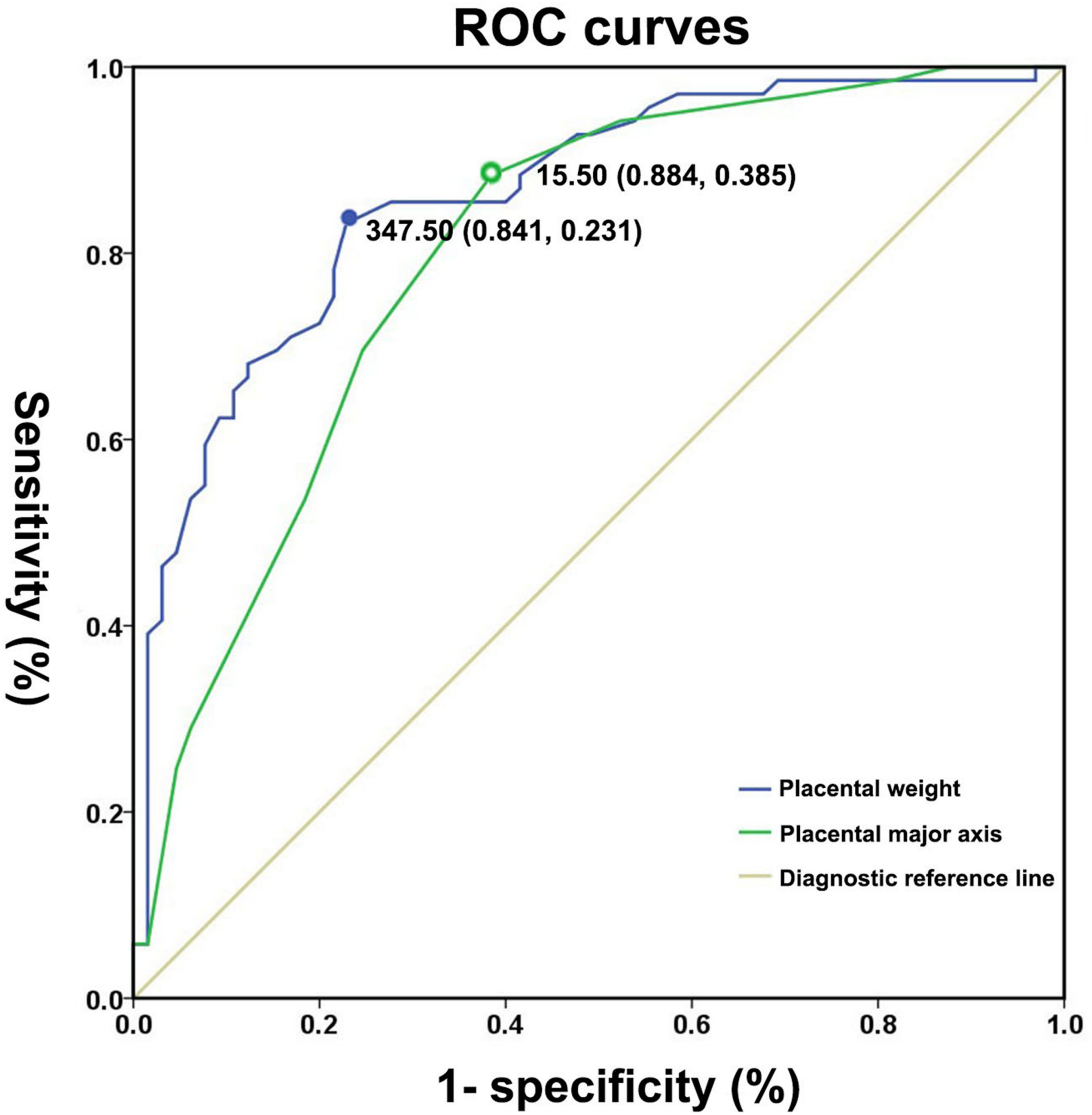
Receiver operating characteristic curves for placental weight and major axis for distinguishing small for gestational age neonates from appropriate for gestational age controls.

ROC curves were used to quantify marker (placental weight and major axis) performance (Figure 3) in the prediction of the subsequent risk of SGA with a mother of PIH. Areas under the ROC curve (AUC) indicated a good discriminatory power of placental major axis (AUC = 0.80, 95% CI 0.72–0.88, *p* < 0.01). With the optimal cut-off of 15.50 cm for the placental major axis, a sensitivity of 88.40% and specificity of 61.50% were achieved in the detection of SGA risk for preterm neonates born to PIH mothers. According to the cut-off value, we divided the subjects into two groups (long placental major axis group ≥15.50 cm and short placental major axis group <15.50 cm). The short placental major axis group tended to have a higher prevalence of SGA [83.33% (40/48) vs. 29.07% (25/86)] and a low percentage of AGA [16.67% (8/48) vs. 70.93% (61/86)] compared to the long placental major axis group.

Areas under the ROC curve (AUC) indicated a good discriminatory power of placental weight (AUC: 0.86; 95% CI: 0.80 − 0.92, *p* < 0.01). The optimal cut-off value of placental weight for predicting the presence of SGA was 347.50 g, with a sensitivity of 84.10% and specificity of 76.90%. According to the cut-off value, we divided the subjects into the two groups (heavy placental weight group ≥347.50 g and light placental weight group <347.50 g). The light placental weight group tended to have higher prevalence of SGA [81.97% (50/61) vs. 20.55% (15/73)] and low percentage of AGA [18.03% (11/61) vs.79.45% (58/73)] compared to the heavy placental weight group.

## 4. Discussion

PIH affects 6%–10% of pregnancies in some developed countries such as Europe and the United States (18). Pregnancy complications are the major causes of perinatal morbidity and mortality in newborns and their mothers (19). PIH-related maternal complications include eclampsia, cerebrovascular disease, abnormal liver and kidney function, cerebral hemorrhage, and placental abruption. Of them, preeclampsia has a significantly higher risk of maternal complications that could seriously affect the life and health of both the fetus and the mother (18). More than half a million fetal and neonatal deaths and 70,000 maternal deaths worldwide are caused by preeclampsia annually (20). The incidence of fetal distress, premature birth, intrauterine growth restriction presenting as SGA neonates, and hematological abnormalities (thrombocytopenia, polycythemia, and neutropenia) have increased in newborns of mothers with PIH, thus requiring prolonged intensive care (14). According to a 3-year retrospective observational study including 6,077 cases, the prevalence of SGA in neonates from mothers with PIH was 31.03%, which is substantially higher than the 2.52% in neonates from healthy mothers (14). In addition, the prevalence of prematurity in neonates from mothers with PIH is 62.07%, which is considerably higher than the 3.58% prevalence in neonates from healthy mothers (14). The placenta is a crucial organ that supports intrauterine life and fetal development (11). Abnormal placental size and shape may be associated with abnormal neonatal birth weight through mismatches between fetal nutritional requirements and placental supply (11, 14). A study examining 1,035 mother-term infant pairs showed that fetal intrauterine growth affected by gestational diabetes, excessive gestational weight gain, and pre-pregnancy obesity was partially mediated by placental weight at birth (21). However, studies reporting placental morphological features or identifying the risk factors related to SGA birth in women with PIH are limited.

The present study suggests that placental size or morphology at birth is strongly correlated with the birth weight of preterm neonates born to mothers with PIH. More specifically, lower placental weight, smaller placental volume and area, shorter major and minor axes, and UC length at birth are risk factors for PIH-complicated fetal growth restriction. Among the above parameters, only short placental major axis and low placental weight were independent risk factors for SGA in premature newborns of mothers with PIH.

In support of the correlation between neonatal weight and placental weight at birth, Ouyang et al. (22) confirmed that the percentile of placental weight at delivery could be used to determine the cause of fetal intrauterine growth restriction. In support of our findings on the association between eventual placental volume and neonatal weight at birth, reduced placental volume as early as the first trimester has been demonstrated in hypertensive disorders of pregnancy associated with SGA fetuses (13). Furthermore, placental volume less than the 10th percentile in the first trimester could be a useful predictor for SGA neonates (23).

Furthermore, the surface area is also a critical placental parameter for fetal development because it determines nutrient transport and oxygen diffusion (24). In this study, we found that PIH-complicated fetal growth retardation was partially associated with a reduced placental area at birth. Ravikumar et al. (11) demonstrated that an eccentric placenta whose minor axis has no apparent length has a smaller placental area and is related to intrauterine growth restriction in SGA infants. Alwasel et al. (25) focused on the relationship between placental shape and fetal growth and demonstrated that placental breadth (minor axis) is positively associated with the placental area, perimeter, and body size at birth. A placenta whose minor axis was close to the major axis (roughly round) had higher placental efficiency. The placental major axis (*p *= 0.025; OR: 2.16; 95% CI: 1.84 − 2.63), not the minor axis, was an independent risk factor for SGA in premature newborns of mothers with PIH in this study. These differences may be attributed to the placenta collected in these studies, which were from hypertension-complicated pregnancies in our study and from pregnancies without comorbidities in the above two studies (e.g., epilepsy, thyroid disease, hypertension, and diabetes mellitus).

Placental thickness is a controversial placental morphological parameter that has been widely investigated in many studies for its correlation with neonatal birth weight. An examination of 561 normal singleton pregnancies throughout pregnancy demonstrated that thick placentas are related to light birth weight and poor perinatal and infant outcomes (26). However, Suri et al. (27) demonstrated that placental thickness in the first trimester was positively related to birth weight in pregnancies with normal outcomes. More interestingly, a prospective cohort study of 991 participants conducted by Vachon-Marceau et al. (28) observed that placental thickness in the first trimester increased in pregnancies accompanied by preeclampsia and decreased in pregnancies at risk of SGA but had no significant change in pregnancies at risk of preeclampsia accompanied with SGA. In this study, we observed similar placental thickness at birth in the SGA and AGA groups, which had the same background of hypertensive risk. These findings suggest that underlying pathologies related to preeclampsia and SGA have opposing effects on early placental growth.

Among the above parameters, short placental major axis and low placental weight were independent risk factors for SGA in premature newborns of mothers with PIH, which were associated with higher SGA delivery risks by 2.16 times and 2.68 times, respectively. As a diagnostic capability, the placental major axis for the prediction of SGA neonates has a sensitivity of 88.40% and specificity of 61.50%, while the placental weight for the prediction of SGA neonates is 84.10% and 76.90% for sensitivity and specificity, respectively. A placental weight lower than 347.50 g or a placental major axis shorter than 15.5 cm at birth was related with a greater risk of delivery of SGA premature infants to mothers with PIH. Our data showed that the placental major axis and low placental weight were reliable clinical indicators for SGA prediction. SGA neonates are more likely to experience adverse outcomes after delivery and even upon reaching adulthood (14). Early recognition of SGA status-related morphological characteristics of the placenta and a high index of suspicion were useful for early intervention and management and for preventing complications (12, 14). As a potential prenatal warning indicator, the placental major axis can easily be obtained by ultrasound before delivery. This advantageous predictive value of the placental major axis requires that more attention is given to it during B-mode ultrasound examinations in the perinatal period. This is likely to provide useful information for perinatal refined pre-evaluation of premature SGA neonates born to PIH mothers. Currently, quantitative observation of the morphological features of the placenta and progress in fetal development has been realized with the development of three-dimensional ultrasonography imaging (14). It can help provide more details regarding the prenatal placenta of SGA infants born to mothers with PIH. In future, studies with larger sample size can develop gestation specific centiles for placental major axis and use it as one of the prenatal parameters in prediction analysis for SGA. The placental major axis, combined with commonly used indicators, such as fetal biparietal diameter and femoral length can be considered in future trials.

The UC is an important link between the fetus and the placenta. Compared with AGA term newborns, thinner (maximum diameter <0.8 cm) UCs were noted in SGA term newborns (12). Other UC abnormalities were also observed in SGA term newborns, including single umbilical artery, knots, hypo- or hyper-coiling, and abnormal insertion (12). However, to date, the association between UC length and birth weight of newborns has rarely been reported. In this study, we found that a shorter UC length was related to reduced neonatal weight at birth in pregnancies complicated with hypertension. Nonetheless, larger sample size studies are still needed to verify these results in the future.

In this study, we demonstrated the detailed morphometric characteristics of the placenta in a study of SGA preterm neonates of mothers with PIH. Furthermore, we revealed that lower placental weight and shorter major axis length were independent risk factors for SGA in premature newborns of mothers with PIH. The predictive value of these two parameters in predicting PIH-related SGA risk was also preliminarily explored. Since the length of the major axis is an easily obtainable ultrasonographic parameter and shows its potential to improve the prediction of the risk of SGA delivery, this research could be useful for monitoring the women with PIH. Furthermore, it could be helpful for the earliest possible intervention and management, not only during pregnancy but also after childbirth, and could prevent adverse neonatal outcomes as much as possible.

Despite its potential clinical importance, this study has several limitations. The results were based on medical records obtained from one institution, and all neonates were Chinese. Due to the limited sample size, a classification study on the placental morphological characteristics of different subtypes of PIH-related SAG was not conducted. Furthermore, owing to the retrospective nature of this study, placental morphological parameters at different pregnancy stages were not collected. This research could be regarded as a preliminary exploration. Prospective multi-center larger cohort studies including neonates of various races should be conducted globally. The findings of this study should also be validated in larger cohorts. Further studies could also precisely determine when placental size and shape start to get affected during pregnancy in mothers with PIH who deliver SGA preterm neonates.

## Data Availability

The raw data supporting the conclusions of this article will be made available by the authors, without undue reservation.
